# Morphine-induced hyperalgesia involves mu opioid receptors and the metabolite morphine-3-glucuronide

**DOI:** 10.1038/s41598-017-11120-4

**Published:** 2017-09-04

**Authors:** Laurie-Anne Roeckel, Valérie Utard, David Reiss, Jinane Mouheiche, Hervé Maurin, Anne Robé, Emilie Audouard, John N. Wood, Yannick Goumon, Frédéric Simonin, Claire Gaveriaux-Ruff

**Affiliations:** 1 Institut de Génétique et de Biologie Moléculaire et Cellulaire, Illkirch, France; 2 Université de Strasbourg, Illkirch, France; 3 Centre National de la Recherche Scientifique, UMR7104 Illkirch, France; 4 Institut National de la Santé et de la Recherche Médicale, U964 Illkirch, France; 5 Biotechnologie et Signalisation Cellulaire, UMR 7242 CNRS Illkirch, France; 6 CNRS UPR3212, Institut des Neurosciences Cellulaires et Intégratives, Centre National de la Recherche Scientifique, Strasbourg, France; 7 Molecular Nociception group, Wolson Institute for Biomedical Research, University College London, WCIE 6BT London, UK

## Abstract

Opiates are potent analgesics but their clinical use is limited by side effects including analgesic tolerance and opioid-induced hyperalgesia (OIH). The Opiates produce analgesia and other adverse effects through activation of the mu opioid receptor (MOR) encoded by the *Oprm1* gene. However, MOR and morphine metabolism involvement in OIH have been little explored. Hence, we examined MOR contribution to OIH by comparing morphine-induced hyperalgesia in wild type (WT) and MOR knockout (KO) mice. We found that repeated morphine administration led to analgesic tolerance and hyperalgesia in WT mice but not in MOR KO mice. The absence of OIH in MOR KO mice was found in both sexes, in two KO global mutant lines, and for mechanical, heat and cold pain modalities. In addition, the morphine metabolite morphine-3beta-D-glucuronide (M3G) elicited hyperalgesia in WT but not in MOR KO animals, as well as in both MOR flox and MOR-Nav1.8 sensory neuron conditional KO mice. M3G displayed significant binding to MOR and G-protein activation when using membranes from MOR-transfected cells or WT mice but not from MOR KO mice. Collectively our results show that MOR is involved in hyperalgesia induced by chronic morphine and its metabolite M3G.

## Introduction

Chronic pain is a major public health problem with a high prevalence and impacting on quality of life. Pain treatments include both opioids and non-opioid analgesics^1^. The mu opioid receptor (MOR) encoded by the *Oprm1* gene is the molecular target for opiate-mediated analgesia and has been shown to be essential for several opiate-induced side effects^2, 3^ such as opioid-induced hyperalgesia (OIH) and analgesic tolerance that preclude adequate analgesia, leaving pain unmanaged^4–6^. However, whether or not OIH requires MOR activation is still an open question. Indeed, some studies have implicated Toll like receptor-4 (TLR4), a key innate immunity receptor, as the mediator for OIH^7–12^. However other studies led to contrasting results^4, 9, 11, 13–15^. Therefore, the clarification of MOR implication in OIH is an important step toward understanding OIH that leads to dose escalation and opioid toxicity. Solving this question will allow designing novel strategies for analgesia and thus provide major improvement to existing pain therapies. It will constitute a first step toward the treatment of OIH itself. It will further impact treatment of addiction, as addiction may be induced by increased pain sensitivity.

In order to determine whether MOR is required for OIH development, we used a genetic approach to compare wild type (WT) and MOR knockout (KO) mice in OIH paradigms. In addition, given that gender represents a major factor for pain and opioid analgesia^16–19^, we studied OIH in both males and females. Then, we determined whether the morphine metabolite morphine-3beta-D-glucuronide (M3G) induces hyperalgesia through MOR activation. To complete the study, we evaluated morphine metabolism to M3G in both genotypes. Morphine- and M3G-induced hypersensitivities were absent in mice harboring *Oprm1* gene inactivation, demonstrating unambiguously that MOR represents a mandatory target for OIH.

## Results

### Chronic morphine induces OIH in WT but not MOR knockout animals

**Figure 1:**
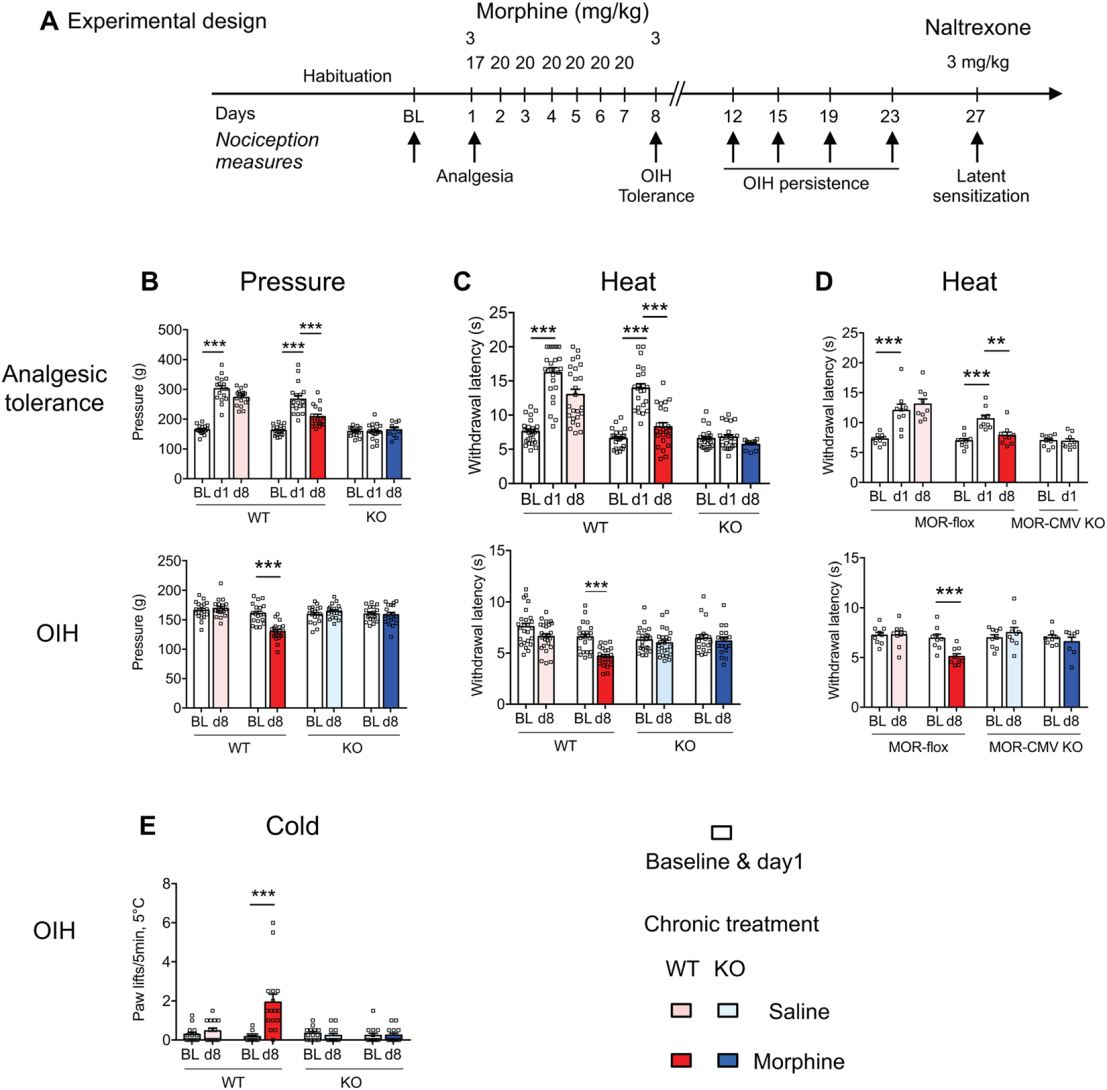
WT but not MOR KO mice show hyperalgesia under repeated morphine analgesic tolerance conditions. (**A**) The experimental design shows the schedule for nociceptive measures (arrows). Following baseline (BL), mice received 3 mg/kg morphine (ip) on day-1 (d1) to evaluate morphine-induced analgesia. Mice received thereafter 20 mg/kg morphine or saline control each day until day-7 (d7). On d8, nociceptive levels were measured before morphine administration to evaluate hyperalgesia, and following 3 mg/kg morphine to measure analgesic tolerance. Maintenance of hyperalgesia (OIH) was scored on the indicated days and latent sensitization on day 27. (**B**) Tail pressure (n = 18–19/group) and (**C**) tail immersion (48 °C, n = 19–25/group) results show analgesic tolerance (upper panels) in WT mice with repeated morphine. Pressure and heat hyperalgesia in WT but not KO mice are shown with the same mouse groups in bottom panels. **p < 0.01; ***p < 0.001 compared to baseline or d1. (**D**) Analgesic tolerance and hyperalgesia occur in MOR-flox but not MOR-CMV mice. **p < 0.01; ***p < 0.001 compared to baseline or to d1 (tail immersion 48 °C, n = 8–9/group). Two-way repeated ANOVA, Newman-Keuls test. **(E**) WT but not MOR KO mice show cold allodynia under morphine analgesic tolerance conditions. Following baseline (BL) cold response scoring on the 5 °C cold plate, mice received 20 mg/kg morphine or saline control once a day until day 7 as described in (A) and paw responses to cold were measured to evaluate cold allodynia. n = 13–16/group. ***p < 0.001 compared to BL. ANOVA repeated measures, Newman-Keuls test. Detailed statistical analyses are presented in Supplementary Table S2.

**Figure 2:**
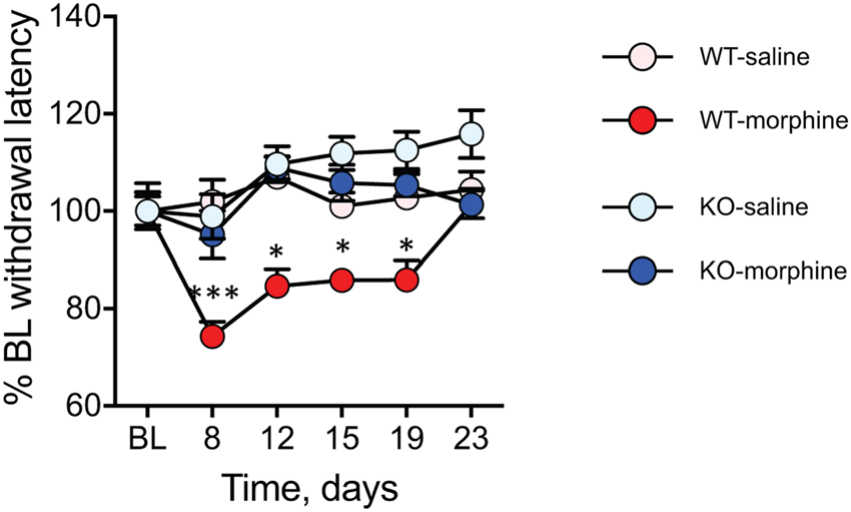
MOR KO mice do not show OIH persistence. Persistence of hyperalgesia after cessation from seven-day 20 mg/kg morphine in WT but not MOR-KO mice. **(B, C)** On day-27 when WT mice had recovered from OIH (Fig. 1A), the opioid antagonist naltrexone induced hyperalgesia *i.e*. latent sensitization in WT (**B**) but no MOR KO (**C**) mice. Tail immersion (48 °C, n = 11–14/group for OIH persistence and n = 6–8/group for latent sensitization following 20 mg/kg morphine; n = 8–10/group for persistence of OIH following 60 mg/kg morphine). **p < 0.01; ***p < 0.001; repeated measures ANOVA followed by Newman-Keuls test. Detailed statistical analyses are presented in Supplementary Table S3.

We first examined whether MOR is required for chronic morphine-induced hyperalgesia by measuring OIH development in WT and MOR KO mice (KO mice with a Neo cassette insertion in exon-2^20^, see Supplementary Fig. S1). As expected, acute morphine induced analgesia in WT mice but not in KO mice (Fig. 1A–C), confirming that *Oprm1* gene-derived MOR is the molecular target for morphine-induced analgesia^2^. The subsequent treatment with repeated morphine (20 mg/kg, 7 days) led to analgesic tolerance as well as OIH in WT mice (Fig. 1A–C). However, no OIH was found in MOR KO mice as assessed for pressure, heat and cold modalities (Fig. 1B,C,E). Next we assessed if OIH persisted following morphine arrest. OIH lasted 11 days upon morphine cessation in WT mice (day 19 of the whole experiment; Fig. 2), in agreement with previous reports^5^. In contrast, no hyperalgesia developed in MOR KO mice after morphine arrest, indicating the absence of late onset hyperalgesia in the mutant animals.

In order to confirm undoubtedly the MOR requirement for OIH, we tested an alternative MOR KO line obtained by crossing MOR-flox mice with CMV-Cre mice and lacking whole *Oprm1* gene exons 2–3 (MOR-CMV mice^21^; Supplementary Fig. S1). The MOR-CMV mice showed neither analgesia nor OIH while control MOR-flox mice responded with analgesia and developed both tolerance and hyperalgesia (Fig. 1D). Then we assessed MOR involvement in OIH by applying another protocol previously used to investigate TLR4 role in morphine analgesic tolerance (60 mg/kg, 4 days^22^). This morphine protocol induced analgesia, tolerance and OIH in WT animals but neither analgesia nor OIH in MOR KO animals (Supplementary Fig. S2). Collectively our results indicate that MOR is essential for morphine-induced analgesia and hyperalgesia independently of the repeated morphine paradigm or of the MOR knockout model used.

### MOR is required for OIH in both females and males

**Figure 3:**
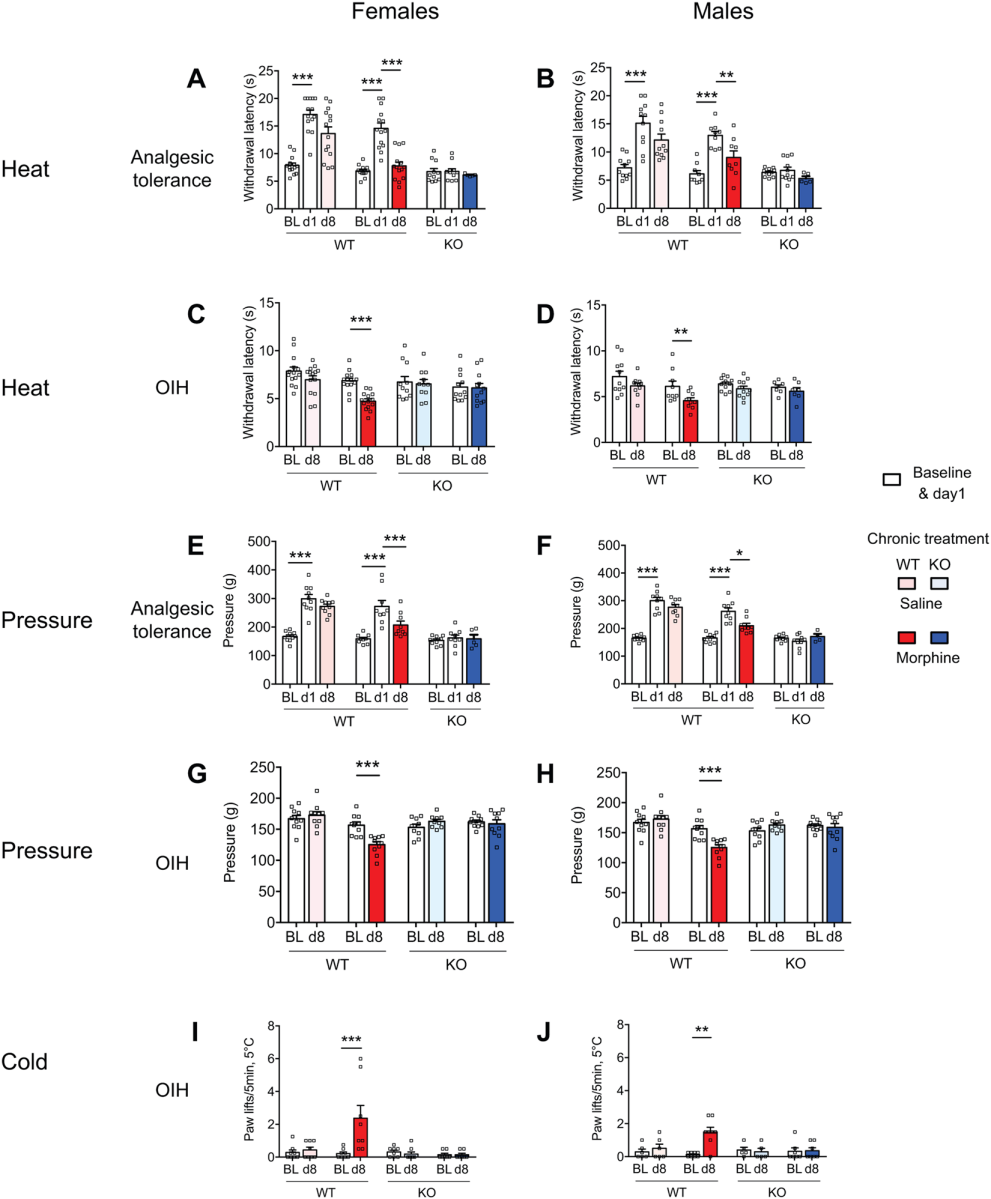
Morphine induced hyperalgesia in female and male WT mice but not MOR KO mice. Analgesia, analgesic tolerance and OIH were measured following the protocol described in Fig. 1A (see sex-grouped analysis in Fig. 1) on female (left panels) and male (right panels) mice with the tail immersion (**A**–**D**), pressure analgesimeter (**E**–**H**) and cold plate (**I,J)** tests for heat, pressure and cold hypersensitivities, respectively. Data are expressed as mean ± SEM. n = 7–11 mice/group. *, **, ***p < 0.05, 0.01 and 0.001 compared to the corresponding group (ANOVA repeated measures, Newman-Keuls. BL, Baseline. Detailed statistical analyses are presented in Supplementary Table S4.

Given that sex represents an important factor that affects pain and analgesia^16, 17^, we have analysed MOR implication in OIH in both the female and male mice. In agreement with previous findings^2^, the absence of MOR did not alter basal sensitivity to heat, cold and mechanical sensitivities, either in females or in males. Morphine elicited comparable analgesia and analgesic tolerance in female and male WT animals and analgesia was absent in KO animals of both sexes (Fig. 3A,B and E,F). OIH induced by chronic morphine developed in WT mice of both sexes, and for all three tested modalities (heat, pressure and cold) and was absent in both female and male MOR KO mice (Fig. 3C,D and G–J), showing no sex-dependency for MOR implication in OIH.

### MOR is required for OIH in neuropathic pain states

**Figure 4:**
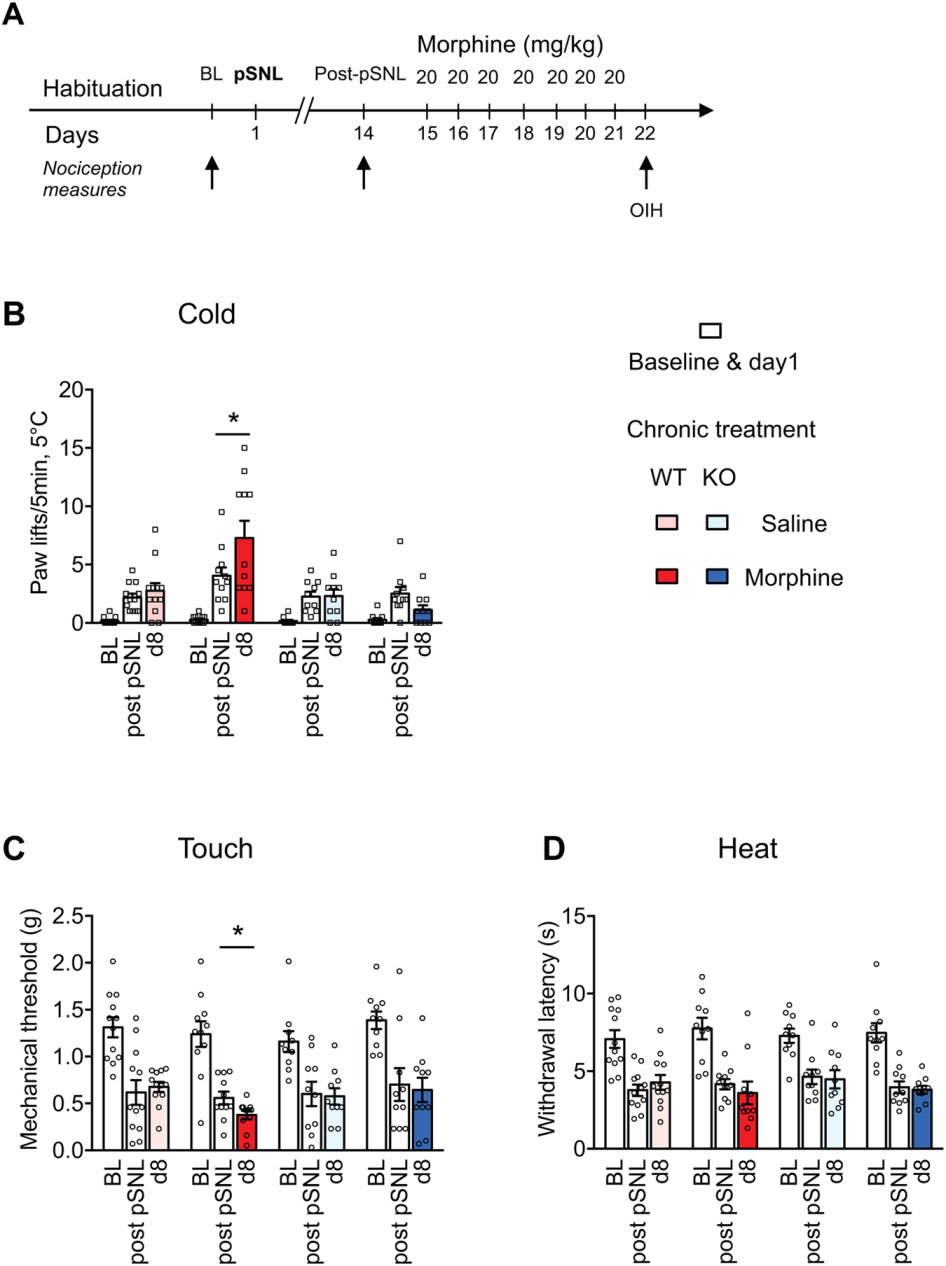
MOR KO mice show no morphine-induced hyperalgesia following partial sciatic nerve ligation (pSNL). (**A**) The experimental design shows the schedule for nociceptive measures (arrows). Following determination of baseline (BL) and neuropathic hypersensitivity (14 days post-pSNL, partial sciatic nerve ligation) on a 5 °C cold plate, WT and MOR KO mice were treated for 7 days with either morphine or saline control solution. On d8, allodynia was measured with the cold plate, von Frey filaments and heat plantar tests. (**B**) Cold pSNL allodynia and OIH (n = 10–12/group), two-way ANOVA, Newman-Keuls test. Morphine induced OIH in WT mice,*p < 0.05 compared to post-pSNL. (**C**) Mechanical pSNL allodynia (n = 10–12/group), two-way ANOVA, Newman-Keuls test. pSNL induced mechanical allodynia; WT saline group and WT morphine group, p < 0.001 pSNL vs BL; KO saline group and KO morphine group, p < 0.01 pSNL vs BL. Morphine induced OIH in WT mice, *p < 0.05 compared to post-pSNL. (**D**) Heat pSNL allodynia (n = 10–12/group), two-way ANOVA. Morphine induced no OIH. pSNL induced cold, mechanical and heat allodynia. Detailed statistical analyses are presented in Supplementary Table S5.

The role of MOR in OIH was further assessed in a neuropathic pain model. Partial sciatic nerve ligation (pSNL) induced cold and mechanical allodynia as well as heat hyperalgesia measured two weeks post-pSNL (Fig. 4). Cold and mechanical allodynia were aggravated by a 7-day repeated morphine treatment in WT mice while no OIH was observed in KO mice (Fig. 4A–C). No OIH developed in WT or KO mice for the heat modality (Fig. 4D), similarly to Corder *et al*.^23^. These results indicate that MOR is also required for OIH after nerve injury.

### MOR KO does not change TLR4 expression

TLR4 activation has been reported as a key event in morphine analgesic tolerance and OIH, although conflicting results have been obtained^4, 24^. Thus, to investigate if the lack of OIH in MOR KO animals may be caused by a difference in TLR4 expression in KO mice, we compared TLR4 transcripts in WT and KO mice. TLR4 expression was comparable in the spinal cord of WT and KO mice treated with chronic saline. Chronic morphine did not alter TLR4 expression in either WT or KO mice (Supplementary Fig. S3). In addition, chronic morphine did not alter MOR expression in WT mice. These findings suggest that the absence of OIH in our MOR KO lines did not result from altered TLR4 expression.

### MOR is necessary for morphine-3-glucuronide hyperalgesia

**Figure 5:**
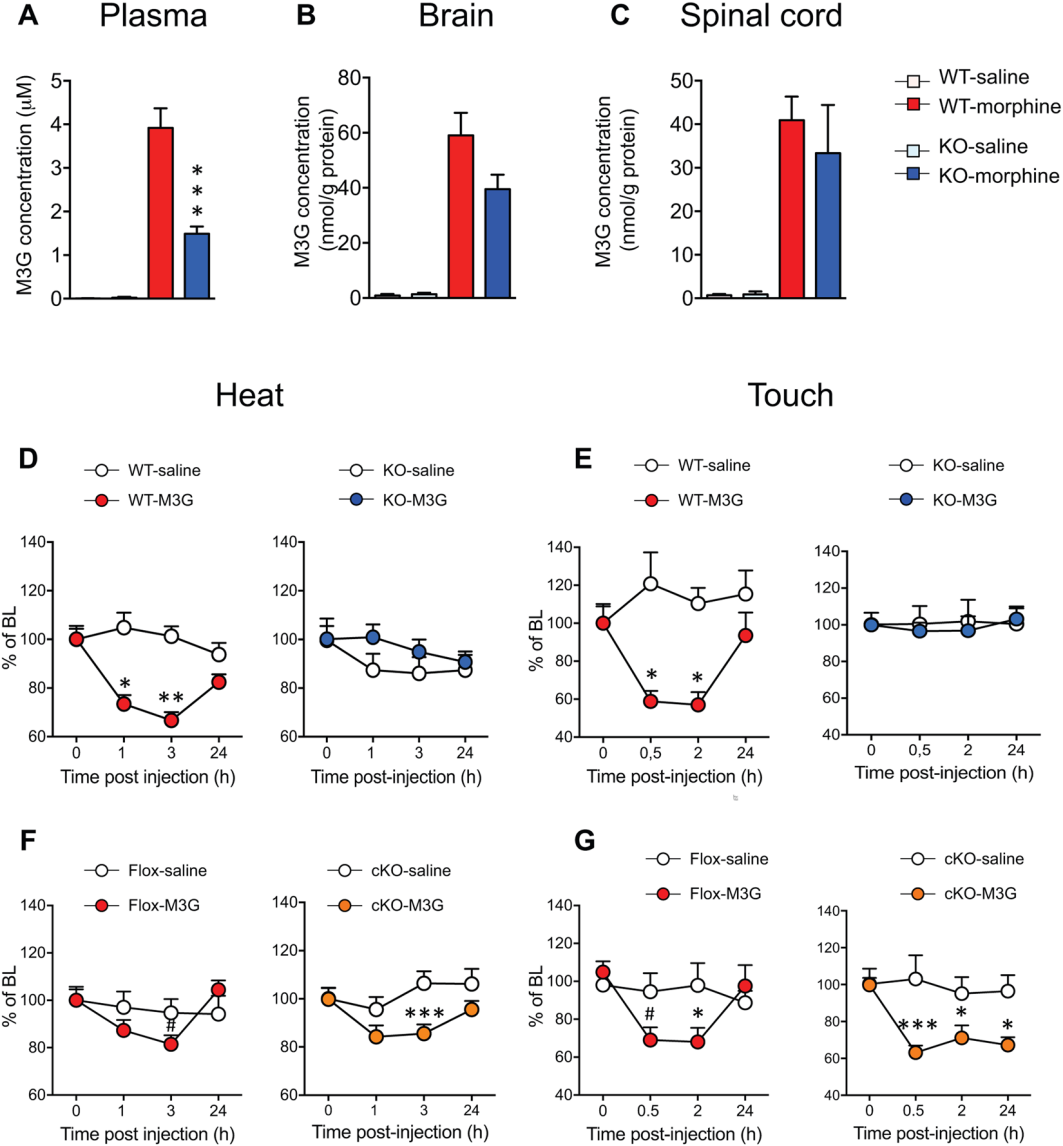
MOR KO mice show no morphine-3-glucuronide-induced hyperalgesia. M3G concentration in mouse plasma (**A**), brain (**B**) and spinal cord (**C**) 2 hr following 10 mg/kg morphine administration. M3G was quantified using LC-MS/MS. Data are expressed as mean ± SEM. n = 9–11 mice/group. ***p < 0.001 in KO mice as compared to WT mice. (**D**,**E**) Acute M3G induces heat and touch hyperalgesia in WT but not KO mice. (n = 9–10/group) one-way ANOVA, Newman-Keuls test *p < 0.05; **p < 0.01 compared to baseline (BL). (**F**,**G**) Acute M3G induces heat and touch hypersensitivity in MOR Flox and cKO mice (n = 6–9/group) one-way ANOVA, Newman-Keuls test #0.05 < p < 0.1; *p < 0.05; ***p < 0.001 compared to baseline (BL). Detailed statistical analyses are presented in Supplementary Table S6.

The main morphine metabolite morphine-3-glucuronide (M3G) has been proposed as a mediator of morphine-induced hyperalgesia^4, 25^. To investigate whether the lack of morphine hyperalgesia in MOR KO animals may be due to an impaired morphine metabolism to M3G in these animals, we measured M3G levels in the plasma, brain and spinal cord following morphine administration. In control saline treated animals, we found very low endogenous M3G levels of 0.0007 μM in WT mice and 0.025 μM in KO mice. In morphine-treated animals, the plasma M3G concentration of the KO mice (1.5 μM) was decreased by 2.6 fold compared to WT mice (3.9 μM), revealing a genotype effect (Fig. 5A). Brain and spinal cord from morphine-treated WT and KO mice also contained significant M3G levels. Brains from KO mice contained 1.5 fold less M3G than those from WTs (Fig. 5B) although the difference was not significant (p > 0.05 Student t-test; n = 10/genotype). Spinal cords from KO and WT mice contained similar M3G levels (Fig. 5C).

To evaluate further if MOR was implicated in M3G-induced hyperalgesia, M3G-induced hypersensitivity was evaluated in WT and MOR KO mice following an acute M3G administration (5 mg/kg i.p.). M3G induced heat hyperalgesia in WT mice within 1–3 hr in the tail immersion assay (Fig. 5D). Similar results were obtained for mechanical hypersensitivity as assessed with the von Frey filaments (Fig. 5E). In contrast, M3G hyperalgesia was lost in KO mice (Fig. 5D,E) revealing that MOR is required for M3G-induced hyperalgesia. Together with morphine metabolism data indicating that KO mice produce M3G, these results suggest that the absence of morphine hyperalgesia in KO mice is not caused by a lack of morphine metabolism to M3G but by a pronociceptive action of M3G through MOR. We have then investigated whether peripheral MOR expressed by Nav1.8 sensory neurons may be involved in M3G hyperalgesia. We observed that conditional KO (cKO) mice deleted for MOR in Nav1.8 neurons displayed comparable hyperalgesia to MOR flox mice (Fig. 5F,G), indicating that this specific MOR population is not mandatory for OIH.

### Morphine-3-glucuronide binds to and activates MOR

**Figure 6:**
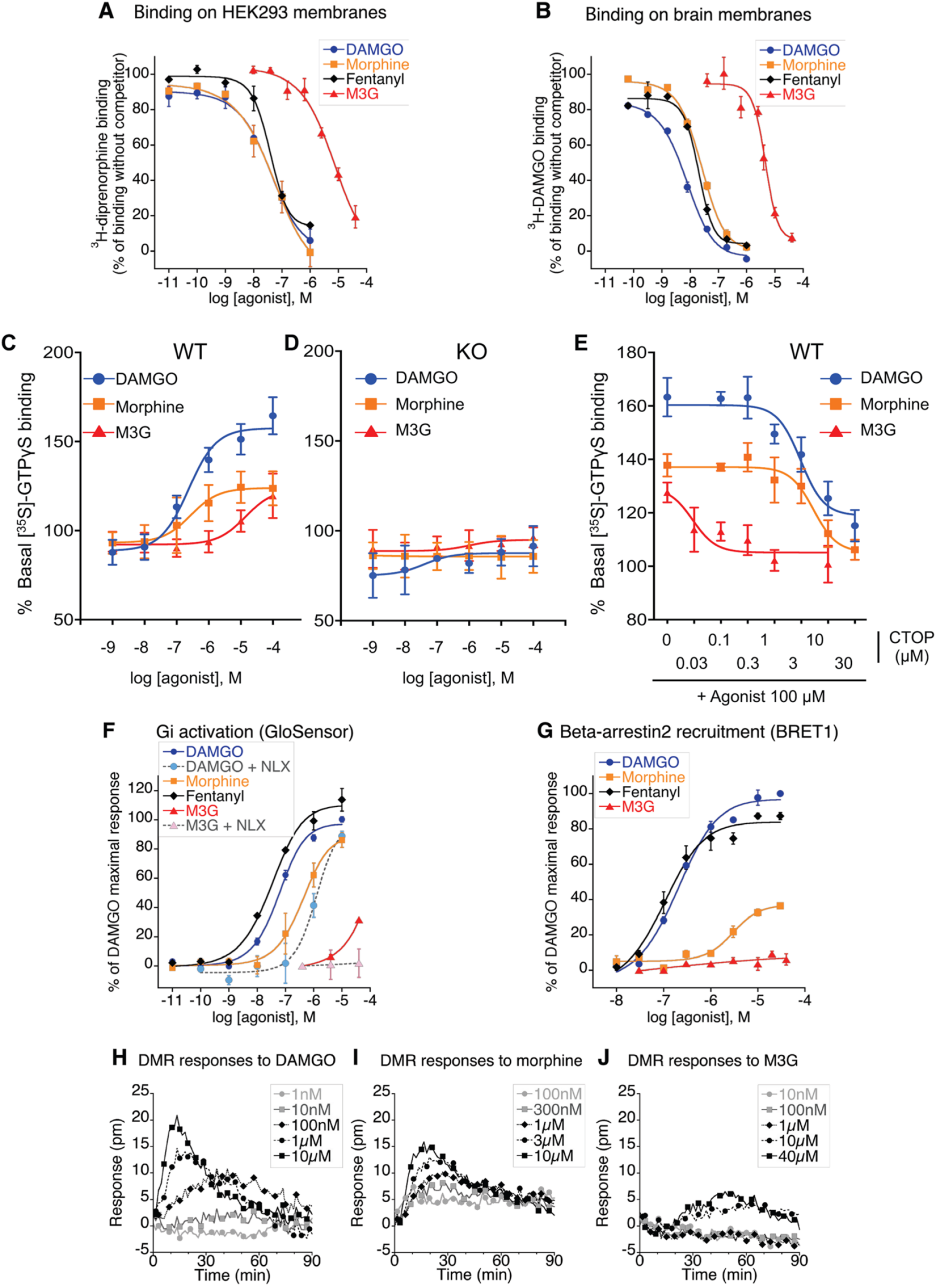
M3G binding and signaling to MOR (**A**,**B)** [^3^H]-diprenorphine binding on membranes from MOR expressing HEK293-Glo cells (**A**) and [^3^H]-DAMGO binding on brain membranes preparations from WT mice (**B**). Membranes were incubated with increasing doses of DAMGO, morphine, fentanyl or M3G in assay buffer containing a fixed dose of opioid radioligand. 100% represents maximal radioligand binding in the absence of competitor. Results are presented as means ± SEM of 2 or 3 experiments. (**C**–**E) M**OR agonist-induced [^35^S]-GTPγS binding to brain membranes preparations from WT (**C**) or KO mice (**D**). Membranes were incubated with increasing doses of DAMGO, morphine or M3G agonists (10^−9^ to 10^−4^M) in assay buffer containing [^35^S]-GTPγS. Basal level (100%) represents [^35^S]-GTPγS binding in the absence of agonist. DAMGO, morphine and M3G significantly stimulated [^35^S]-GTPγS binding to membranes from WT but not KO mice. Results are presented as means ± SEM of 3–5 experiments on 3 independent membrane preparations per genotype. (**E**) The selective MOR antagonist CTOP inhibits DAMGO, morphine and M3G-induced [^35^S]-GTPγS binding to membranes from WT mice. Brain membranes from WT mice were incubated with increasing doses of the mu opioid antagonist CTOP combined with 100uM of DAMGO, morphine or M3G. Activation results are presented as means ± SEM of 6–10 experiments from 3 independent membrane preparations. (**F**) Effect of increasing concentrations of DAMGO, morphine, fentanyl and M3G on forskolin-stimulated cAMP production in HEK293 stably expressing GloSensor and MOR receptor. In the cases indicated, 1 µM of naloxone had been added to the cells 15 min prior to the agonist. Dose−response curves were normalised to maximal DAMGO activity. Evaluations were performed three times in duplicate for M3G, two times in duplicates for other agonists. Results are presented as means ± SEM. **(G)** eYFP-labelled Beta-arrestin-2 translocation to Rluc-MOR in HEK293 cells after 5 to 10 min of cells activation by DAMGO, morphine, fentanyl or M3G at 37 °C. Agonist specific BRET1 ratio were determined by subtracting BRET1 ratio of non activated cells, and normalised to maximal DAMGO-triggered effect. Presented results are means ± SEM of 2 to 4 experiments. **(H–J)** Dynamic mass redistribution (DMR) signals observed in HEK293-Glo-cells after activation by various concentrations of DAMGO, morphine or M3G. Baseline of buffer-treated cells has been subtracted. Evaluations were performed three times in duplicate or triplicates. Presented figures show a representative experiment.

Morphine-3-glucuronide has been characterized as a weak **partial** MOR agonist in transfected cells^25^. We first investigated M3G binding to membranes from MOR-transfected HEK293 cells. M3G displaced [^3^H]-diprenorphine binding, although with a lower affinity as compared to cold DAMGO, morphine or fentanyl (Supplementary Table S1, Fig. 6A). In addition, M3G displaced [^3^H]-DAMGO binding to brain homogenates from WT mice. The affinity for M3G-induced displacement of [^3^H]-DAMGO binding was 1.4 µM as compared to nanomolar affinities for DAMGO, morphine and fentanyl (Supplementary Table S1, Fig. 6B), indicating that M3G binds to MOR receptor expressed in brain cells. No specific [^3^H]-DAMGO binding was observed in brain homogenates from MOR KO mice (data not shown). We then determined whether M3G could activate MOR by comparing G-protein activation in brain from WT and MOR KO mice. We used the [^35^S]-GTPγS binding assay on brain membranes in which MOR activation provokes [^35^S]-GTPγS binding to G-proteins. Morphine and DAMGO MOR-selective agonists as well as M3G activated MOR in WT brain membranes (Supplementary Table S1, Fig. 6C). In contrast, none of the agonists induced [^35^S]-GTPγS binding to KO brain membranes (Fig. 6D). The blockade of M3G-evoked [^35^S]-GTPγS signal to WT membranes by the MOR selective antagonist CTOP confirmed the MOR selectivity (Supplementary Table S1, Fig. 6E). Therefore our results on the selective activation of MOR by M3G corroborate *in vivo* MOR-dependent M3G hyperalgesia. We further investigated M3G-induced MOR signalling in HEK-293 cells stably expressing MOR. We observed that M3G displayed a weak MOR agonist activity on adenylate cyclase (Supplementary Table S1), which was blocked by naloxone (Fig. 6F), as well as in a label free assay of dynamic mass redistribution (DMR; Fig. 6J). However, the peak of M3G action in DMR assay appeared to occur later than with DAMGO and morphine (Fig. 6H,I), suggesting that this morphine metabolite could activate slightly different signalling pathways than classical opioid agonists. Moreover, we observed no beta-arrestin2 recruitment in MOR-HEK293 cells with M3G, while DAMGO, fentanyl and morphine (although with a lower potency) displayed significant beta-arrestin2 recruitment (Supplementary Table S1; Fig. 6G). Altogether, our data indicate that M3G significantly interacts with and signals through MOR *in vitro* although with a weak potency, and implicate MOR for OIH *in vivo*.

## Discussion

Our results obtained with two different MOR KO mouse lines and two repeated morphine schedules reveal that MOR is required for morphine-induced hyperalgesia. They confirm and extend other findings obtained with different repeated morphine administration schedules^26^. The lack of OIH in global KO mice was revealed here by using tail immersion, pressure and cold plate assays for heat, mechanical and cold hypersensitivities while in this previous study^26^ tail immersion, hot plate and von Frey filaments have been employed. Additionally, we show MOR requirement in OIH under a neuropathic condition using cold and mechanical sensitivity tests. Corder *et al*.^26^ did not observe OIH when assessing heat and mechanical modalities following morphine administered 7 days post-unilateral chronic construction injury. We could detect OIH in WT mice receiving repeated morphine starting 14 days post-pSNL when testing for cold and touch hypersensitivity, but not for heat response. This suggests that the cold and mechanical sensitivity may be more affected by chronic morphine in neuropathic condition, and OIH was absent in nerve-injured MOR KO mice. Altogether this indicates that MOR is mandatory for OIH in both basal and neuropathic conditions.

It has been proposed that analgesia and OIH may be induced by specific isoforms of MOR, such as a 6TM (6-transmembrane domain) isoform, discovered since the initial MOR characterization as a 7TM receptor ^27–29^. Indeed, the activation of the 6TM MOR-1K isoform lacking the N-terminal region and first TM domain induces excitatory cellular effects by activating Gs while activation of the 7TM receptor inhibits neurons via Gi activation^30^, relating the 6TM hypothesis to earlier works on the excitatory mechanisms for OIH^31, 32^. Moreover spinal silencing of the 6TM MOR-1K variant in CXB7/ByJ mice led to decreased morphine hyperalgesia, suggesting that the Gs coupling of this 6TM MOR form could be important for OIH development^29, 33^. Our study shows that OIH is abolished in both MOR KO mice harboring a Neo cassette insertion in exon-2^20^ and MOR-CMV-KO mice lacking TM2-7^21^ and so lacking all TM7 and TM6 potential isoforms. This indicates that TM2-7 are necessary for morphine-induced hyperalgesia and leaves open 7TM and 6TM mechanisms for OIH.

Our results document that OIH developed similarly in both female and male WT mice and was absent in MOR KO mice of both sexes, showing no sex-influence for MOR implication in OIH under our experimental conditions. Similarly, we found no sex difference for basal nociception and morphine analgesia. Sex is known to be an important factor influencing pain and analgesia although dependent upon genetic and environmental factors^16, 17, 34^. Furthermore, sex-dependent involvement of spinal microglia and T lymphocytes have been shown to control inflammatory and neuropathic hypersensitivity^35, 36^. Previously OIH was shown to be more pronounced in female than male rats when using low sub-analgesic morphine doses^37^ while in morphine-infused mice there was no sex difference^38^, or the sex difference depended on morphine dose and genetic background^39, 40^. We can conclude that under our experimental conditions the role of MOR in OIH investigated through the comparison of WT and KO animals was apparent in both female and male mice, strengthening the conclusion on MOR implication. Along with congruent recent findings^26^ and previous studies that indicated some downstream pathways associated with OIH and exhibiting MOR dependency (see in ref. 4, 5), our results demonstrate that TLR4 is not the sole mandatory receptor in OIH.

Opioid-induced hyperalgesia may be distinguished from withdrawal-induced pain in that withdrawal reaction is produced by the abrupt cessation of opioid administration whereas OIH is a state of pain sensitivity that may last longer. We have shown the MOR requirement in the long-term OIH as hyperalgesia lasts for 12 days after morphine arrest in the non-neuropathic pain paradigm and is abolished in the KO mice (Fig. 2). In the neuropathic model, hypersensitivity was investigated only on the day post morphine cessation. Whether this hyperalgesia that follows nerve injury and chronic morphine treatment would be withdrawal-induced hyperalgesia, or rather long-lasting OIH caused by activation of opponent nociceptive processes, remains to be determined as well as the common and distinct molecular and cellular adaptations underlying the two types of hypersensitivity responses. Multiple mechanisms have been shown to contribute to both opioid-induced hypersensitivities^4–6, 41, 42^. They include amongst others the pronociceptive actions of low morphine levels and of opioid metabolites, excitatory mechanisms in MOR-expressing cells and at some sites of the pain control system as well as pronociceptive anti-opioid and neuroimmune processes. One cause of OIH was shown to be due to low residual morphine levels following morphine cessation, as acutely administered low morphine levels similar to those found two days after cessation of morphine produced hyperalgesia^43^. This mechanism was described to be opioid-dependent as OIH was reversed by the opioid antagonist naloxone^43^, and may thus contribute to OIH in our experiments. Morphine-3-glucuronide is the main morphine metabolite and was reported to mediate hyperalgesia. The plasma concentrations of morphine and M3G peak at 30 min and 3 hrs post-administration in mice^44^ and humans^45^, respectively. Morphine and M3G levels remain stable upon chronic morphine administration^46^, indicating that chronic treatments do not lead to morphine or M3G accumulation that would trigger pain. Our data reveal that acute M3G decreases nociceptive levels, at a lower dose than previously described^9^. In addition, we show that MOR is required for M3G-induced hyperalgesia, suggesting that M3G-MOR may mediate OIH.

Both MOR and TLR4 have been identified as OIH targets for morphine and M3G^4, 5, 47^, and our present data indicate that MOR is required for M3G-induced hyperalgesia. Morphine-3-glucuronide has been characterized as a weak MOR ligand and partial agonist. It displaces [3 H]-DAMGO binding to guinea-pig brain homogenates with a Ki of 0.36 μM as compared to the Ki of 0.0018 μM reported for morphine^48^. A weak affinity, as assessed by [3 H]-naloxone binding displacement to human MOR expressed in HEK293 cells, was shown (Ki 6 μM), together with an 8 μM EC50 for Gi activation as compared to 0.05 μM for DAMGO and 0.015 μM for morphine and a bias toward b-arrestin2 recruitment as compared to morphine in MOR-transfected HEK293 cells^25, 49^. Our study confirmed the weak affinity for binding to MOR expressed both on MOR-transfected HEK293 cells and in mouse brain and indicate that M3G is a partial agonist as it is unable to elicit a maximal 100% response in several signaling assays.

Following morphine administration, micromolar M3G concentrations are found in blood (our results and ref. 44). We recorded similar M3G levels in brain and spinal cord of WT and KO animals that can activate MOR to produce hyperalgesia in the WTs. The cause for the lower M3G levels in the plasma of KO mice than plasma from WT mice, while M3G contents were similar in WT and KO brain and spinal cord, may be further explored. The difference in plasma M3G may be due to alterations in morphine metabolism^44^ by glucuronyl transferases^50^, pharmacokinetics, passage through the blood-brain barrier through ATP-binding cassette transporters^51^ or organic anion transporting polypeptides^52^, as well as elimination^44^. Altogether our results suggest that some differences in M3G signalling compared to other opioid agonists could explain why this molecule displays hyperalgesic rather than analgesic activity. Besides, M3G was shown to display low affinity binding to TLR4 and to induce cellular effects distinct from the classical TLR4 activator LPS. In the HEK-Blue-TLR4 Secreted alkaline phosphatase (SEAP) signalling assay, M3G weakly activates TLR4 but also partly inhibits LPS-induced TLR4 activation^24^. Furthermore M3G does not elicit classical LPS-induced activation markers in human and mouse macrophages^24^, while morphine effects on macrophages were reported to be MOR-dependent and independent^53, 54^. In addition, the investigation of TLR4 involvement in OIH by using TLR4 knockout mice led to conflicting results^4, 9, 11, 13, 14^.

We analysed spinal TLR4 expression level in WT and KO animals and found comparable TLR4 levels in WT and MOR KO animals, indicating that MOR gene inactivation does not alter TLR4 expression in a major way. In addition, chronic morphine did not elevate TLR4 levels in either WT or MOR KO mice, showing that OIH does not require TLR4 up-regulation while not excluding potential MOR-TLR4 interactions in OIH. Interestingly, morphine elevated TNF-α expression in macrophages in mice, and TNF-α was shown to stimulate NF-kB activation and SEAP production in a TLR4-independent manner in the HEK-Blue-TLR4 reporter cells, implicating macrophage TNF-α as a novel mediator for OIH^55^. Also, morphine was found to activate NF-kB in a MOR-dependent fashion in microglia^56^ and to increase Brain-Derived Neurotrophic factor (BDNF) in microglia in a TLR4-independent way^13^. In contrast, spinal microglia were found to be activated in MOR KO mice displaying no OIH after morphine treatment, suggesting no correlation between spinal microglia activation and OIH^26^. Nonetheless, in this study the spinal microglia activation state was not documented in MOR KO mice in the absence of morphine and other papers show Oprm1 expression in microglia^57, 58^, precluding a definitive conclusion on this point.

Recently, a novel OIH mechanism dependent on MOR expressed by nociceptors has been shown, based on targeted *Oprm1* gene inactivation in Trpv1-Cre positive cells^26^. OIH is absent in these MOR-Trpv1 cKO mice^26^ while we find a maintained OIH in MOR-Nav1.8 cKO mice. Collectively this suggests that MOR on Trpv1-positive/Nav1.8-negative cells is implicated in OIH. In the DRGs, Nav1.8 and Trpv1 neurons only partially overlap. Lagerström *et al*.^59^ has shown that Nav1.8-Cre + DRG neurons include 67% Trpv1-expressing neurons while Trpv1 neurons comprise 76% Nav1.8-expressing cells. Also, based on single-cell transcriptomics, Usoskin *et al*. have classified DRG neurons into 11 eleven types^60^. MOR is expressed in one subclass of Nav1.8-high-Trpv1-low neurons, in one subclass of Trpv1-high-Nav1.8-low neurons, and in three subclasses of double Nav1.8-Trpv1 neurons belonging to the peptidergic or non-peptidergic neurons. MOR displays also a low expression in one subclass of neurofilament-positive neurons. The role of MOR expressed by these different subclasses of peripheral neurons needs to be further investigated. Mechanisms for OIH also include descending pain facilitation from rostral ventromedial medulla (RVM)^61^. Finally, neuron-microglia interactions in reward and other central circuits were shown important for injury-induced pain and tolerance^62^ and might in that respect participate in OIH.

In conclusion, morphine-induced hyperalgesia was absent in MOR KO mice, highlighting MOR requirement on OIH. Taken together, the present and previous studies suggest a role for specific MOR populations^26, 61, 63^, isoforms^33^ or signalling^64^ at some nervous system sites that require further investigation.

The MOR-dependent mechanisms may involve the morphine metabolite M3G as M3G-hyperalgesia is also lost in MOR KO animals. Furthermore, our results on MOR in OIH clearly show that TLR4 is not the sole OIH mediator, and the relationships between these two receptors need to be further explored. Targeting biased signalling at MOR^65^ may help designing novel therapeutic analgesic strategies devoid of opioid side effects including OIH. Finally, the crucial role of MOR shown here justifies strategies based on MOR delta-opioid receptor heterodimers^66^, MOR-chemokine receptor and MOR beta-2-adrenergic receptor cross talk^41, 67^, or putative MOR - TLR4 functional interactions to attenuate analgesic tolerance and OIH.

## Methods

### Animals and ethical statement

All the experiments were carried out in accordance with the European Communities Council Directive of 22 September 2010 (directive 2010/63/UE), under the guidelines of the Committee for Research and Ethical issues of IASP published in PAIN, 1983; 16:109-110 and were approved by the local ethical committee (Com’Eth, Comité d’Ethique pour l’Expérimentation Animale IGBMC-ICS, licence N° 17) with the agreement number 00876-02. Mice were housed under standard light, temperature and humidity conditions (12 h light-dark cycle, 21 ± 1 °C, 55 ± 10% humidity). Cage bedding was from Anibed (Pontvallain, France; reference AB3) and food from SAFE (Augy, France; reference D03). Water (autoclaved tap water) was available ad libitum. Mice were kept group-housed at 2–4/cage. In each group equal numbers of male and female mice were used, aged of 10–16 weeks. Mice were habituated to their experimental environment and handled for one week before starting the experiments. Particular efforts were made to minimize the number of mice and the pain they experienced. Behavioral tests were performed blind to the genotype and treatment. Studies are reported following the ARRIVE Guidelines for reporting experiments involving animals^68^. Mice lacking mu opioid receptors (conventional MOR KO mice^20^) had been generated by homologous recombination as described in Supplemental Fig. 1A. These were compared to their wild type (WT) littermates for behavioral and molecular studies. Behavioral results were analyzed on grouped males and females except for the female-male separate analysis shown in Fig. 3. We used a second MOR KO mouse line lacking whole *Oprm1* gene exons 2–3, obtained by crossing MOR-flox mice with CMV-Cre mice^21^, see Supplementary Fig. 1C. These mice are named MOR-CMV throughout the study. MOR-CMV KO mice were compared to their Oprm1-gene floxed littermates (MOR-flox mice) in experiments shown in Fig. 1D. The genetic background of all mice was 50% C57/BL6J:50% 129svPas.

### Behavioral testing

Nociception assays were performed as previously described^21^. To determine heat nociceptive thresholds, the tail immersion test was performed by immersing half of the tail in a 48 °C water-bath. The latency until tail withdrawal was recorded, with a 20 s cut-off latency to avoid tissue damage. Cold allodynia was determined with the cold plate apparatus (Bioseb, Vitrolles, France). Each mouse was placed on the plate (5 °C) for a 5 min period, and the number of paw lifts was counted. Nociception to pressure was measured with the Pressure Application Measurement device (Ugo Basile, Milano, Italia). Briefly, a progressive pressure was applied manually onto the tail and pressing was stopped when a nociceptive reaction was observed. The pressure value at the reaction time was automatically recorded. Sensitivity to touch was determined by using Von Frey filaments applied under the hind paw of the mouse and following the Up and down method as described^21^.

### Drugs and treatments

Morphine chlorhydrate (Francopia, Gentilly, France) in saline solution (NaCl 0.9%) or saline control solution were injected intraperitoneally (ip) using 100 μl solution per 10 g mouse body weight. Three different repeated morphine administration protocols were used. The first one is shown in Fig. 1A. Following baseline nociception measures, all mice received 3 mg/kg morphine on day 1 (d1) to induce analgesia. Once analgesia has been scored, mice were randomly distributed into 2 groups, the saline control group and the morphine group injected on the first day with 17 mg/kg morphine (to complete the dose up to 20 mg/kg) or saline, and on the 6 following days with 20 mg/kg morphine. The last day (d8), before morphine injection, nociceptive thresholds were determined in order to measure morphine hyperalgesia and then, mice received a morphine dose (3 mg/kg) to evaluate morphine analgesic tolerance. In the second protocol (see Supplementary Fig. S2), mice received 60 mg/kg repeated morphine once a day during 4 days. The overall design of the 2 protocols is similar, with morphine injections at 3 mg/kg on d1 and d8 or d5 to measure analgesic tolerance, respectively. In both protocols, nociceptive thresholds were scored on d5 or d8, before last morphine dose, to evaluate morphine-induced hyperalgesia. Morphine acute effects (analgesia and analgesic tolerance) were measured 45 min after 3 mg/kg morphine administration, and hyperalgesia was measured 20–22 hours following daily morphine (20 or 60 mg/kg). At the end of both repeated morphine treatments, mice were tested with tail immersion test (+d4, +d7, +d11, and +d15 post-treatment) to measure the persistence of hyperalgesia (Fig. 2A). In both protocols, basal nociceptive levels were recovered at +d15 post morphine cessation.

The third protocol was assayed on neuropathic mice (Fig. 4). Neuropathic pain was induced by partial ligation of the left common sciatic nerve (pSNL) by 7–0 braid silk suture under ketamine/zylazine anesthesia (100/10 mg/kg mixture; ketamine, Virbac, Carros, France; xylazine, Rompun, Bayer Healthcare, Puteaux, France) according to the method described previously^69^. Nociceptive reactions at baseline and on day 14 post-pSNL were scored as described above. From day 15, the mice were then administered 20 mg/kg morphine or the saline control solution for 7 consecutive days. Cold, heat and mechanical sensitivities were scored the next morning following the last morphine dose (d8) for OIH assessment.

For the analysis of morphine metabolism, mice were injected ip with 10 mg/kg morphine or 100 μl saline control solution, and tissues collected 2 hr later.

Morphine-3-beta-D-glucuronide (M3G; Sigma Aldrich, St Louis, USA) at 5 mg/kg in saline or a saline control solution were injected ip in naive mice. Hypersensitivity induced by M3G was measured by using the Von Frey test (0.5, 2 and 24 hr post-injection) and the tail immersion test (48 °C; 1, 3 and 24 hr post-injection), respectively as described above.

Animals were allocated to experimental groups according to gender (male or female) and genotype. Littermates of the same sex were randomly assigned to the experimental groups. The number of animals per group was designed in accordance with previous similar studies^21, 70^. The results shown were obtained from 2–4 cohorts/group/experiment.

### Preparation of plasma and tissues for M3G determination by liquid chromatography-tandem mass spectrometry (LC-MS/MS)

Plasma was prepared from blood collected in lithium-heparin tubes (BD, ref 367526) by centrifugation at 1300 × g for 15 min. Supernatant was collected into low-binding microtubes. Brains and spinal cords were homogenized with an Ultra Turrax (Ika, Staufen, Germany) in 1 ml of H_2_O, respectively. The homogenates were then sonicated (2 times 10 s, 90 W) with a Vibra Cell apparatus (Sonics, Newtown, USA) and centrifuged (14,000 × g, 30 min). Supernatant was recovered and the concentration of proteins was determined using the Bradford method (Protein Assay, Bio-Rad, Marnes-la-Coquette, France). In order to quantify morphine and M3G in brain and spinal cord (300 µl) of each extract were acidified with 500 µl of 0.5% formic acid (v/v). Plasma (100 µl) was acidified with 500 µl of 0.5% formic acid (v/v). After centrifugation (14,000 × g, 15 min, 4 °C), supernatants were collected prior to solid phase extraction (SPE). The SPE procedure was performed with a positive pressure manifold (Thermo Electron, Courtaboeuf, France). HyperSep PGC SPE-cartridges (1cc, 25 mg, Thermo Electron) were first activated with 1 ml of acetonitrile (ACN) and then washed with 2 ml of H_2_O/formic acid 0.1% (v/v). Samples were loaded on SPE-cartridges. Cartridges were dryed 1 min under vacuum, and were washed with 1 ml of H_2_O/formic acid 0.1% (v/v). Prelution was performed with 1 ml of ACN 2%/H_2_O 97.9%/formic acid 0.1% (v/v/v). Elution was performed with 800 µl of ACN 20%/H_2_O 79.9%/formic acid 0.1% (v/v/v). Eluates were then collected in low binding 1.5 ml tubes and centrifuged (14,000 × g, 10 min, 4 °C). Supernatants were dried under vacuum prior to MS analysis (see below). Eluates were resuspended in 100 µl H_2_O/formic acid 0.1% (v/v) and 10 µl was injected on the LC-MS/MS.

### Quantitative LC-MS/MS instrumentation and analytical conditions

LC-analyses were used to determine the presence of morphine, M3G in the selected reaction monitoring mode (SRM). Analyses were performed on a Dionex Ultimate 3000 HPLC system (Thermo Scientific, San Jose, CA, USA) coupled with a triple quadrupole Endura (Thermo Scientific). The system was controlled by Xcalibur 2.0 software (Thermo Scientific). Samples were loaded into a column Accucore C18 RP-MS column (ref. 17626–102130; 100 × 2.1 mm 2.6 μm, Thermo Scientific) heated at 40 °C. Dryed samples were dissolved in 100 μl of 0.1% formic acid (v/v) and 10 µl of solution was injected. Elution were performed at 400 µl/min of buffers A/B. Buffer A corresponded to ACN 1%/H_2_O 98.9%/formic acid 0.1% (v/v/v), whereas buffer B was ACN 99.9%/formic acid 0.1% (v/v). After 3 min of 1% of buffer B, a linear gradient of 1–60% of solvent B was applied over 8 min and followed by a washing step (1 min at 99% of solvent B) and an equilibration step (1 min of 1% of buffer B). Qualitative analysis and quantification were performed in SRM. For ionization, 3500 V of liquid junction voltage and 342 °C capillary temperature were applied. The selectivity for both Q1 and Q3 was set to 0.7 Da (FWHM). The collision gas pressure of Q2 was set at 2 mTorr of argon. For morphine and M3G, the selection of the monitored transitions and the optimization of the collision energy were manually determined. The transitions and the corresponding collision energies (CE) used were the following: m/z 286.2 → m/z 147.0 (CE = 57 eV), m/z 286.2 → m/z 173.1 (CE = 46 eV), m/z 286.2 → m/z 201.1 (CE = 38 eV) for morphine; m/z 462.2 → m/z 286.2 (CE = 49 eV) for M3G. m/z 289.2 → m/z 155.1 (CE = 33 eV), m/z 289.2 → m/z 165.2 (CE = 39 eV), m/z 289.2 → m/z 201.1 (CE = 25 eV) for d3-morphine; m/z 465.2 → m/z 289.2 (CE = 30 eV) for d3-M3G. Identification of the compounds was based on precursor ion, selective fragment ions and retention times obtained for deuterated internal standards (d3-morphine and d3-M3G).

### Radioligand binding assays

WT or KO mice brains were homogenized in 10 volumes ice-cold 50 mM Tris/1 mM EDTA/0.25 M sucrose (pH 7.4) and centrifuged at 4 °C for 10 min at 500 g. Supernatants were centrifuged at 4 °C for 15 min at 100,000 g and the pellets were suspended in 5 volumes of 50 mM Tris/1 mM EDTA/0.25 M sucrose buffer (pH 7.4). Aliquots were kept at −80 °C until use. For binding assays, 80 µg of proteins were incubated for 1 h at 25 °C with 3.5 nM [^3^H]-DAMGO (Perkin Elmer, Boston, USA) and increasing concentrations of compounds to be tested, in a final volume of 0.5 mL of 50 mM Tris/1 mM EDTA assay buffer (pH 7.4). Non-specific binding was defined in the presence of 10 µM naloxone. Membrane-bound radioactivity was separated from free radioligand by rapid filtration on Whatman GF/B glass fiber filters, using a Brandel harvester. Radioactivity was quantified by liquid scintillation counting using a TRI-CARB Packard counter.

Membranes from HEK293 cells selected for stable expression of both GloSensor and human MOR were obtained as previously reported^71^. 20 µg of membrane proteins were incubated (1 h at 25 °C) with 0.7 nM [^3^H]-diprenorphine (PerkinElmer) and compounds to be tested, in a final volume of 0.2 mL of 50 mM Tris/1 mM EDTA buffer (pH 7.4). Non-specific binding was defined with 1 µM naloxone. After rapid filtration using a Unifilter-96 Filtermate Cell Harvester (Perkin Elmer), membrane-bound radioactivity was counted in a TopCountNTX microplate counter (Packard).

Data were analyzed using Kaleidagraph software (Synergy Software, Reading, PA, USA). Two or three independent assays were performed in duplicates. Specific binding was converted in percentage of the maximal specific binding without competitor, defined at 100%. IC50s were converted in Ki values using the Cheng-Prusoff equation.

### cAMP accumulation assay

cAMP responses were examined by using the GloSensor^TM^ cAMP assay according to manufacturer recommendations (Promega, Madison WI, USA) with a few modifications. Stable HEK293-Glo-MOR cells were suspended (10^6^ cells per ml) in physiological Hepes buffer (10 mM HEPES, 0.4 mM NaH_2_PO_4_, 137.5 mM NaCl, 1.25 mM MgCl_2_, 1.25 mM CaCl_2_, 6 mM KCl, 5.6 mM glucose and 1 mg/ml bovine serum albumin, pH 7.4) supplemented with 1 mM d-Luciferin (Synchem UG & Co., Felsberg, Germany). Following a luciferin-loading time of 2 h at 25 °C, 100,000 cells per well were distributed in white 96-well plates. Kinetic recordings of the luminescence level were acquired using a FlexStation III microplate reader (Molecular Devices, Sunnyvale, USA) at 25 °C: Hepes buffer or 1 µM naloxone were added at time t = 10 min, various concentrations of agonists were injected at t = 25 min, 0.125 µM of forskolin (Sigma Aldrich) was added at t = 35 min and readings were pursued for 2 h. The presence of 0.5 mM 3-isobutyl-1-methylxanthine prevented cAMP degradation by phosphodiesterases. Two to four independent assays were performed in duplicates and data were analyzed using Kaleidagraph software to provide EC50 values.

### Beta-Arrestin-2 recruitment

Assay was done as described^72^ with a few modifications. Briefly, HEK293 cells stably expressing eYFP-tagged beta-arrestin-2 were transfected with a plasmid encoding Rluc8-MOR. One day after transfection, cells were seeded in white 96-well plates and grown for one more day. Culture medium was replaced by Hepes buffer on the assay day (see cAMP accumulation assay description). After 40 min equilibration at 37 °C, 5 µM Coelenterazine H (ThermoFisher Scientific, Illkirch, France) was added (10 min before BRET1 end-point recording), followed by compounds to be tested (5 min before BRET1 signal recording). Signals were acquired in a VictorLight apparatus (Perkin Elmer) at 37 °C. A “BRET ratio” corresponding to the signal in the “acceptor channel” (band-pass filter 510–560 nm) divided by the signal in the “donor channel” (band-pass filter 435–485 nm) was calculated. Drug-induced BRET was determined (BRET1 ratio of drug-activated cells minus BRET1 ratio of buffer-treated cells) and normalised to the maximum of DAMGO-induced BRET, defined as 100%. Two or three independent experiments were performed in duplicates and data were analyzed using Kaleidagraph software to provide EC50 values.

### Dynamic Mass Redistribution (DMR) assay

The DMR assay was performed on stable HEK293-Glo-MOR cells as described^73^. Cells were seeded (30 µL per well of a 300,000 cells/mL suspension) onto a Cellular Label-free 384-well microplate (Perkin Elmer) previously coated with collagen from rat tail (Sigma Aldrich) and containing 10 µL medium per well. Plate was let in a hood at room temperature for 30 min before overnight incubation à 37 °C in a humidified CO_2_ incubator. The day of the assay, four careful washes of the cell layer with Hepes buffer (see “cAMP accumulation” description) were done, after which the plate was let to equilibrate with 30 µL per well Hepes buffer for 2 h in a EnSpire 2300 Multimode Plate Reader (Perkin Elmer). DMR was then monitored in the apparatus at room temperature before and after compound addition (10 µL added per well). Three independent experiments were done in duplicate or triplicates. For results representation, kinetic curves of control conditions (buffer-treated cells) were subtracted.

### Agonist-stimulated [^35^S]-GTPγS binding assay

DAMGO ([D-Ala2, N-MePhe4, Gly-ol]-enkephalin; Sigma Aldrich, St Louis, USA), morphine, M3G and CTOP ([H-D-Phe-Cys-Tyr-D-Trp-Orn-Thr-Pen-Thr-NH2]; Sigma Aldrich, St Louis, USA) were used in [^35^S]-GTPγS binding assay to measure G protein activation following receptor stimulation. Brain membranes were prepared from WT and conventional KO mice as previously described^21^. Membrane preparations from brain were incubated for 1 h at 25 °C with increasing concentrations (10^−9^ to 10^−4^ M) of agonists (DAMGO, Morphine or M3G) in the assay buffer containing 30 µM GDP and 0.1 nM [^35^S]-GTPγS (NEG030H, PerkinElmer, Courtaboeuf, France). Basal [^35^S]-GTPγS binding was determined in the absence of agonist, and non-specific binding by replacing [^35^S]-GTPγS by cold GTPγS. For the experiment with the mu antagonist CTOP, WT brain membranes were incubated with a fixed dose of DAMGO, morphine and M3G (10^−4^M) and with increasing concentrations of CTOP (0 to 30 μM) to assess the specific activation of mu-receptor by these agonists. Stimulated specific binding was converted in percentage of basal specific binding, defined as 100%. Data were analyzed using Prism 6 Graphpad software. Four to ten independent assays were performed on three distinct membrane preparations per genotype. Stimulation (%), EC50s and IC50s were calculated for each experiment and averaged.

### Quantitative RT-PCR

Mouse cohorts independent from those used for the behavioral tests were treated with chronic morphine or saline as described in Supplemental Fig. 3A. Spinal cords (L4-L6) were collected on d5 18 h after the last 60 mg/kg morphine dose. They were deeply frozen in liquid nitrogen and stored at −80 °C. Quantitative RT-PCR was performed as described^70^. Briefly, total RNA was extracted with TRIzol (Invitrogen, Cergy Pontoise, France). RNA concentration was determined with a ND-1000 Nanodrop spectrophotometer and 1 µg of total RNA was reverse-transcribed in a final volume of 20 µL. Real time PCR was done in triplicate on cDNA with the Light-Cycler-480 (Roche, Mannheim, Germany). To determine hypoxanthine-guanine phosphoribosyltransferase (*HPRT*), mu opioid receptor (*Oprm1*) and Toll like Receptor 4 (*TLR4)* transcript expression levels, the following primers were used: GGTCCTTTTCACCAGCAAGCT (*HPRT* forward), TGACACTGGTAAAACAATGCA (*HPRT* reverse); GAGCCACAGCCTGTGCCCT (*Oprm1* forward), CGTGCTAGTGGCTAAGGCATC (*Oprm1* reverse); AAGAACATAGATCGAGCTTCAACCC (*TLR4* forward), GCTGTCCAATAGGGAAGCTTCTAGAG (*TLR4* reverse). Relative expression ratios (TLR4 in KO saline *vs* in WT saline; TLR4 in WT morphine *vs* in WT saline; TLR4 in KO morphine *vs* in KO saline and Mu in WT morphine *vs* in WT saline) were calculated by using *HPRT* as the reference gene and the 2^−ΔΔCt^ method to determine gene expression levels.

### Statistical analysis

All data are presented as mean ± SEM. Statistical analyses were performed using the Statistica 12 software (StatSoft, Tulsa OK, USA). For behavioral studies one, two or three-way repeated measures ANOVAs were performed followed by Newman-Keuls *post hoc* analysis. Results for persistence of hyperalgesia and M3G induced hyperalgesia are represented as % of baseline. RT-qPCR data were analyzed for individual group differences with a one-way ANOVA. *P* < 0.05 was considered significant.

### Data availability statement

All data generated or analysed during this study are included in this published article (and its Supplementary Information files).

## Electronic supplementary material

Supplementary information
