# Supplementary material for: Morphine-induced hyperalgesia involves mu opioid receptors and the metabolite morphine-3-glucuronide
Source: Sci Rep. 2017 Sep 4;7:10406. doi: 10.1038/s41598-017-11120-4 (PMC5583172; doi:10.1038/s41598-017-11120-4)
Supplement: Supplementary file 1 — Supplementary information [file 41598_2017_11120_MOESM1_ESM.pdf]

Supplementary Figures and Tables

Morphine-induced hyperalgesia involves mu opioid receptor and the metabolite morphine-3-glucuronide

Laurie-Anne Roeckel, Valérie Utard, David Reiss, Jinane Mouheiche, Hervé Maurin, Anne Robé, Emilie Audouard, John N. Wood, Yannick Goumon, Frédéric Simonin and Claire Gaveriaux-Ruff

Table S1. Comparison of M3G and other MOR agonists in MOR-radioligand binding and signaling assays

Compound	[3H]-diprenorphine binding to hMOR in HEK293 cells	[3H]-DAMGO binding to WT mouse brain membranes	[35S]-GTPγS binding to WT mouse brain membranes		Inhibition of cAMP production in hMOR HEK293 cells	Beta-arrestin-2 recruitment in hMOR HEK293 cells	
	Ki, nM	Ki, nM	EC_{50}, nM	CTOP IC_{50}, nM	EC_{50}, nM	EC_{50}, nM	% of DAMGO effect
DAMGO	9.6 ± 0.1	3.1 ± 0.4	265 ± 82	3140 ± 1202	62 ± 10	198 ± 6	100
Fentanyl	8.6 ± 0.8	5.8 ± 1.0	ND	ND	40 ± 10	127± 31	84
Morphine	14 ± 7	7.7 ± 0.9	415 ± 208	3705 ± 1372	422 ± 196	2722 ± 557	37
M3G	1272 ± 166	1403 ± 91	14,299 ± 3276	133± 23	> 40,000	Und	Und

ND, not determined ; Und, undetectable

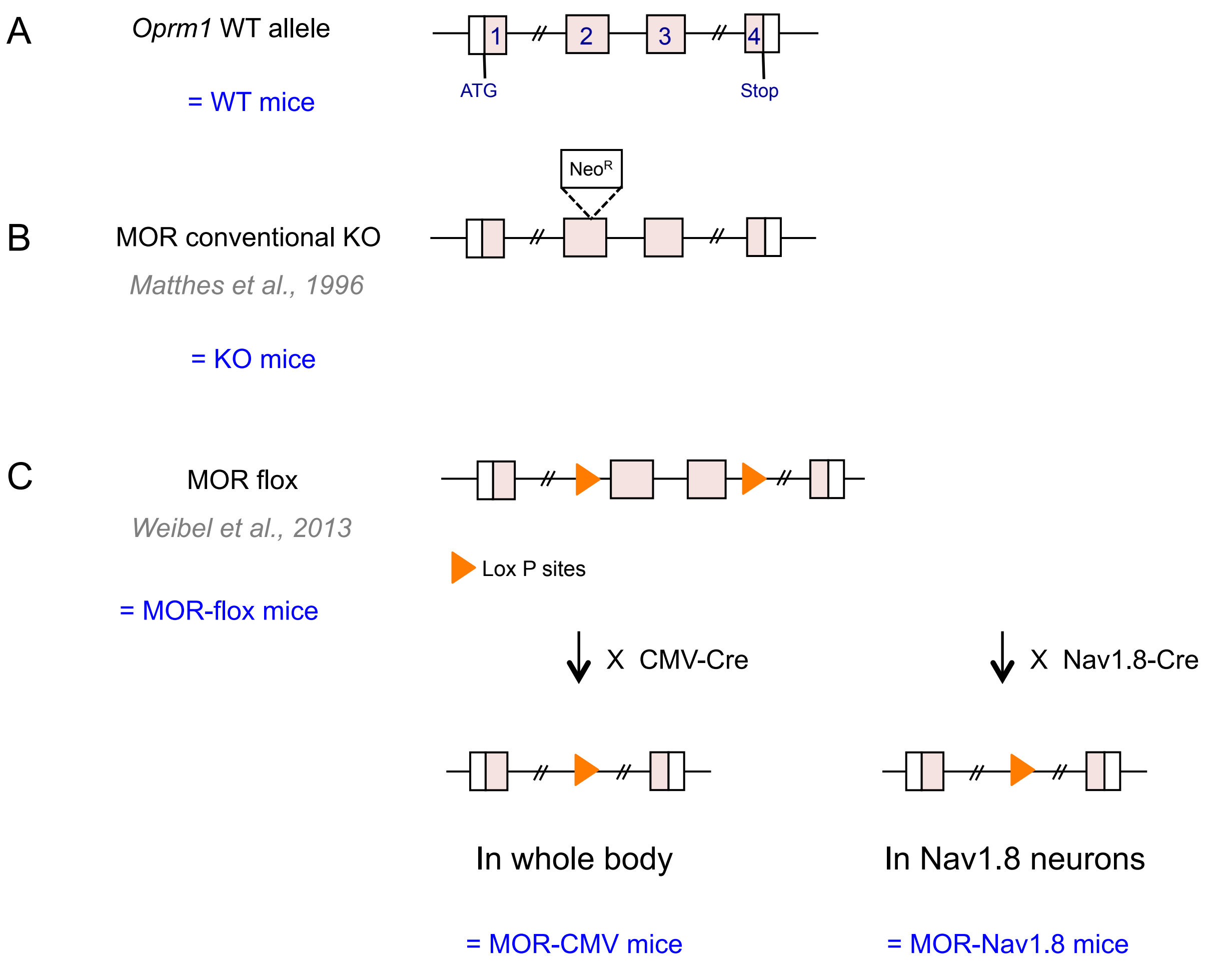

Supplementary Figure S1. The different MOR mutant mouse lines used in the study. (**A**) *Oprm1* gene WT allele. (**B**) For generating MOR conventional KO mice, a neomycin resistance cassette (Neo^R) had been inserted in exon 2, causing the receptor gene inactivation. (**C**) Crossing of mice harboring floxed exons 2-3 with mice expressing the cyclic recombination enzyme (Cre) DNA under the Cytomegalovirus (CMV) promoter control led to ubiquitous MOR gene KO. Crossing with Nav1.8-Cre mice led to Oprm1 gene inactivation in peripheral Nav1.8 neurons. The abbreviations used throughout the text and figures are written in blue characters. CMV, cytomegalovirus ubiquitous promoter; KO, knockout; MOR, mu opioid receptor; *Oprm1*, gene encoding MOR.

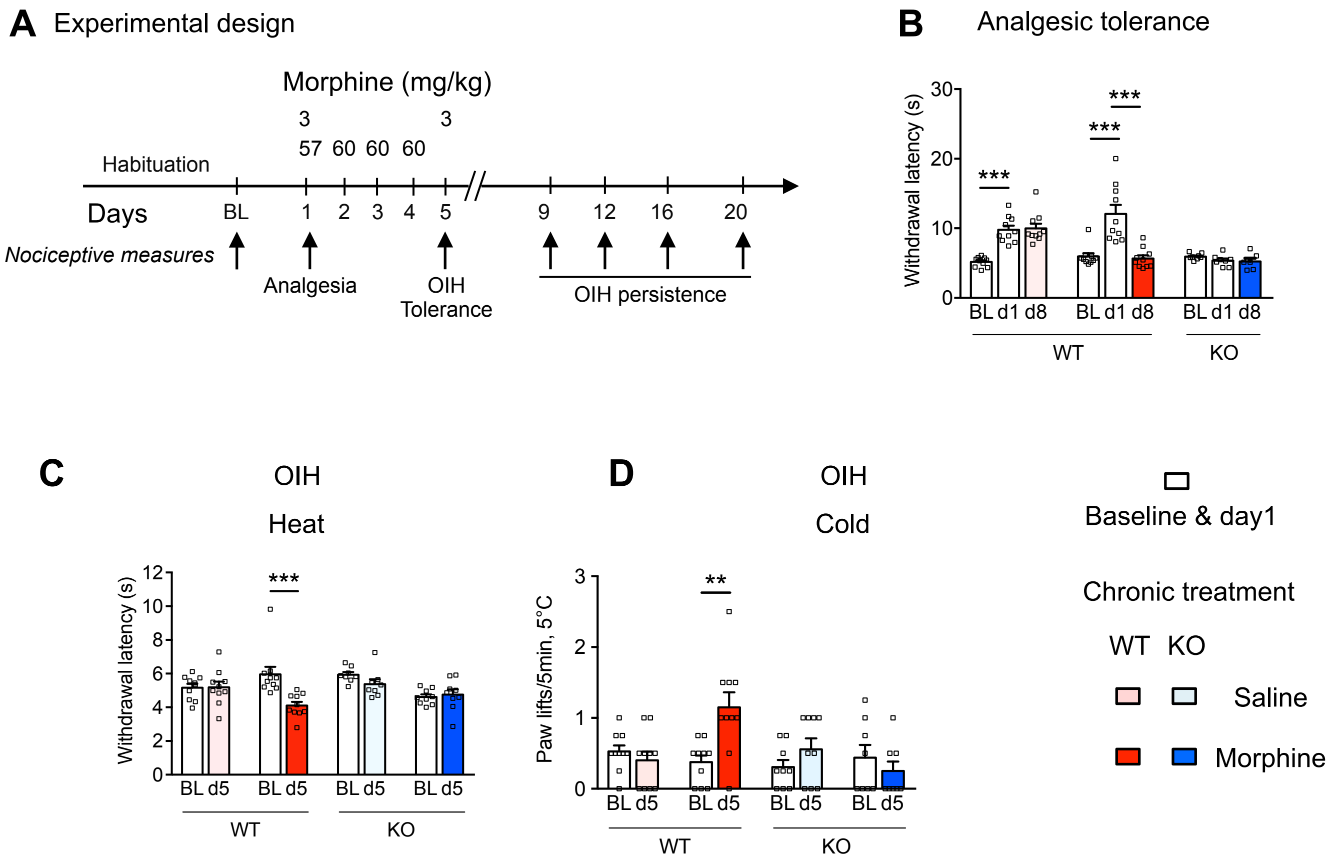

Supplementary Figure S2. Morphine induced hyperalgesia in WT but not MOR KO mice following a repeated 4 day 60mg/kg treatment. Analgesia, analgesic tolerance and OIH were measured following the protocol described in (**A**). (**B**) Analgesia and analgesic tolerance in the 48°C tail immersion test. (**C**) Heat hyperalgesia in the same test. (**D**) Cold allodynia measured with the 5°C cold plate test. **, *** p<0.01, p< 0.001 compared to the corresponding group (ANOVA repeated measures, Newman-Keuls. BL, Baseline. Data are expressed as mean ±SEM. n=7-10 mice/group. Detailed statistical analyses are presented in Supplementary Table S7.

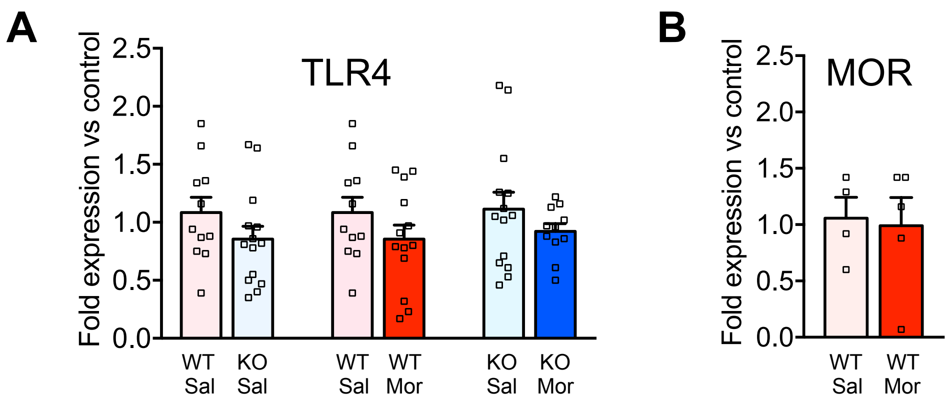

Supplementary Figure S3. There is no difference for TLR4 transcript expression in KO mice and WT mice. Mice were treated with repeated morphine (60 mg/kg, 4 days; Mor) or a saline control (Sal) solution following the protocol described in Supplementary figure S2. RNA was extracted from spinal cord, and TLR4 and MOR transcripts quantified by RT-qPCR. (**A**) MOR KO or morphine induced no change in TLR4 mRNA expression. (**B**) Repeated morphine did not change MOR expression as compared to saline controls. Data are expressed as mean ±SEM. n = 4-10 mice per group. One-way ANOVA. Detailed statistical analyses are presented in Supplementary Table S8.

Supplementary Table S2. Detailed statistical evaluation for Fig. 1.

One-way ANOVA repeated measures for analgesia and tolerance to analgesia

WT	Pressure
Treatment	F(1,35)=29.751, p<0.001
Time	F(2,70)=124.521, p<0.001
Time x Treatment	F(2,70)=8.216, p<0.001

KO	Pressure
Treatment	F(1,35)=0.64, p=0.42
Time	F(2,70)=0.38, p=0.53
Time x Treatment	F(2,70)=0.80, p=0.37

WT	Heat
Treatment	F(1,46)=19.053, p<0.001
Time	F(2,88)=108.213, p<0.001
Time x Treatment	F(2,88)=5.186, p<0.001

KO	Heat
Treatment	F(1,39)=0.76, p=0.38
Time	F(2,78)=1.457, p=0.23
Time x Treatment	F(2,78)=2.098, p=0.12

MOR-flox	Heat
Treatment	F(1,16)=13.99, p<0.001
Time	F(2,32)=23.301, p<0.001
Time x Treatment	F(2,32)=7.785, p<0.01

CMV-KO	Heat
Treatment	F(1,15)=0.44, p=0.51
Time	F(2,30)=0.046, p=0.83
Time x Treatment	F(2,30)=1.477, p=0.24

Two-way repeated measures ANOVA for hyperalgesia

WT vs KO	Mechanical
Genotype<br>Treatment<br>Genotype x Treatment	F(1, 66)=139.98, p<0.001<br>F(1, 66)=16.17, p<0.001<br>F(1, 66)=13.34, p<0.001
Time<br>Time x Genotype	F(2,132)=149.55, p<0.001<br>F(2,132)=160.37, p<0.001

Time x Treatment<br>Time x Genotype x Treatment	F(2,132)=3.29, p<0.05<br>F(2,132)=2.12, p=0.12

WT vs KO	Heat
Genotype<br>Treatment<br>Genotype x Treatment	F(1, 85)=0.36, p= 0.54<br>F(1, 85)=12.18, p<0.001<br>F(1, 85)=4.69, p<0.05
Time<br>Time x Genotype<br>Time x Treatment<br>Time x Genotype x Treatment	F(1, 85)=45.262, p<0.01<br>F(1, 85)=20.104, p<0.01<br>F(1, 85)=2.460, p=0.12<br>F(1, 85)=4.652, p<0.05

MOR-flox vs MOR-CMV KO	Heat
Genotype<br>Treatment<br>Genotype x Treatment	F(1, 31)=1.43, p= 0.24<br>F(1, 31)=6.10, p<0.05<br>F(1, 31)=2.43, p=0.12
Time<br>Time x Genotype<br>Time x Treatment<br>Time x Genotype x Treatment	F(1, 31)=7.672, p<0.01<br>F(1, 31)=9.656, p<0.01<br>F(1, 31)=15.540, p<0.001<br>F(1, 31)=4.121, p=0.051

WT vs KO	Cold
Genotype<br>Treatment<br>Genotype x Treatment	F(1, 53)=9.47, p<0.01<br>F(1, 53)=5.22, p<0.05<br>F(1, 53)=6.14, p<0.05
Time<br>Time x Genotype<br>Time x Treatment<br>Time x Genotype x Treatment	F(1, 53)=13.029, p<0.001<br>F(1, 53)=17.334, p<0.001<br>F(1, 53)=13.354, p<0.001<br>F(1, 53)=8.734, p<0.01

Supplementary Table S3. Detailed statistical evaluation for Figure 2.

Two-way repeated measures ANOVA for persistence of hyperalgesia following cessation from 20mg/kg chronic morphine

WT vs KO	Heat
Genotype	$F(1, 45)=21.67$, $p<0.001$
Treatment	$F(1, 45)=6.29$, $p<0.05$
Genotype x Treatment	$F(1, 45)=24.30$, $p<0.001$
Time	$F(4, 180)=1.568$, $p=0.18$
Time x Genotype	$F(4, 180)=3.505$, $p<0.01$
Time x Treatment	$F(4, 180)=2.88$, $p<0.05$
Time x Genotype x Treatment	$F(4, 180)=0.997$, $p=0.41$

Supplementary Table S4. Detailed statistical evaluation for Fig. 3.

Two-way ANOVA repeated measures for analgesia and tolerance to analgesia in WT

WT	Heat
Treatment	F(1,44)=18.20, p<0.001
Gender	F(1,44)=2.04, p=0.16
Treatment x Gender	F(1,44)=0.675, p=0.41
Time	F(2,88)=108.67, p<0.001
Time x Gender	F(2,88)=1.25, p=0.160.29
Time x Treatment	F(2,88)=5.186, p<0. 01
Time x Gender x Treatment	F(2,88)=0.99, p=0.37

WT	Pressure
Treatment	F(1,33)=28.72, , p<0.001
Gender	F(1,33)=0.002, p=0.96
Treatment x Gender	F(1,33)=0.014, p=0.90
Time	F(2,66)=118.702, p<0.001
Time x Gender	F(2,66)=0.163, p=0.84
Time x Treatment	F(2,66)=7.86, p<0. 001
Time x Gender x Treatment	F(2,66)=0.223, p=0.80

Three-way repeated measures ANOVA for hyperalgesia

WT vs KO	Heat
Treatment	F(1, 81)=13.50, p<0.001
Gender	F(1, 81)=1.10, p=0.29
Genotype	F(1, 81)=86.100, p<0.001
Treatment x Gender	F(1, 81)=0.08, p=0.77
Genotype x Treatment	F(1, 81)=3.38, p=0.06
Gender x Genotype	F(1, 81)=1.109, p=0.29
Gender x Genotype x Treatment	F(1, 81)=0.034, p=0.89
Time	F(1,81)=42.401, p<0.001
Time x Gender	F(1,81)=0.038, p=0.84
Time x Treatment	F(1,81)=2.169, p=0.14
Time x Genotype	F(1,81)=17.321, p<0.001
Time x Genotype x Treatment	F(1,81)=3.510, p=0.06
Time x Gender x Treatment	F(1,81)=0.439, p=0.50
Time x Genotype x Gender x Treatment	F(1,81)=0.479, p=0.49

WT vs KO	Pressure
Treatment	F(1, 66)=16.17, p<0.001
Gender	F(1, 66)=0.000, p=0.95
Genotype	F(1, 66)=139.98, p<0.001
Treatment x Gender	F(1, 66)=0.01, p=0.92
Genotype x Treatment	F(1, 66)=13.34, p<0.001

Gender x Genotype	F(1, 66)=0.00, p=0.95
Gender x Genotype x Treatment	F(1, 66)=0.42, p=0.52
Time	F(2,132)=149.55, p<0.001
Time x Gender	F(2,132)=0.46, p=0.63
Time x Treatment	F(2,132)=3.29, p<0.05
Time x Genotype	F(2,132)=160.37, p<0.001
Time x Genotype x Treatment	F(2,132)=2.12, p=0.12
Time x Gender x Treatment	F(2,132)=0.26, p=0.76
Time x Genotype x Gender x Treatment	F(2,132)=1.14, p=0.32

WT vs KO	Cold
Treatment	F(1, 49)=4.92, p<0.05
Gender	F(1, 49)=0.08, p= 0.72
Genotype	F(1, 49)=9.19, p<0.01
Treatment x Gender	F(1, 49)=0.46, p= 0.49
Genotype x Treatment	F(1, 49)=5.98, p<0.01
Gender x Genotype	F(1, 49)=1.24, p= 0.27
Gender x Genotype x Treatment	F(1, 49)=1.38, p= 0.24
Time	F(1,49)=12.48, p<0.001
Time x Gender	F(1,49)=0.49, p=0.48
Time x Treatment	F(1,49)=12.81, p<0.001
Time x Genotype	F(1,49)=16.89, p<0.001
Time x Genotype x Treatment	F(1,49)=8.18, p<0.01
Time x Gender x Treatment	F(1,49)=0.61, p=0.43
Time x Genotype x Gender x Treatment	F(1,49)=1.01, p=0.32

Supplementary Table S5. Detailed statistical evaluation for Fig. 4.

Two-way repeated measures ANOVA for chronic morphine induced hyperalgesia in WT and KO mice under pSNL neuropathic injury.

WT vs KO	Cold
Genotype	$F(1, 39)=11.25$, $p<0.01$
Treatment	$F(1, 39)=5.47$, $p<0.05$
Genotype x Treatment	$F(1, 39)=9.09$, $p<0.01$
Time	$F(2,78)=34.814$, $p<0.001$
Time x Genotype	$F(2,78)=9.283$, $p<0.001$
Time x Treatment	$F(2,78)=1.886$, $p=0.15$
Time x Genotype x Treatment	$F(2,78)=6.816$, $p<0.05$

WT vs KO	Heat
Genotype	$F(1, 38)=0.19$, $p=0.66$
Treatment	$F(1, 38)=0.47$, $p=0.49$
Genotype x Treatment	$F(1, 38)=0.12$, $p=0.72$
Time	$F(2,76)=78.766$, $p<0.001$
Time x Genotype	$F(2,76)=0.191$, $p=0.82$
Time x Treatment	$F(2,76)=0.386$, $p=0.68$
Time x Genotype x Treatment	$F(2,76)=1.654$, $p=0.19$

WT vs KO	Mechanical
Genotype	$F(1, 39)=0.53$, $p=0.46$
Treatment	$F(1, 39)=4.43$, $p<0.05$
Genotype x Treatment	$F(1, 39)=0.01$, $p=0.92$
Time	$F(2,78)=51.542$, $p<0.001$
Time x Genotype	$F(2,78)=0.167$, $p=0.84$
Time x Treatment	$F(2,78)=0.236$, $p=0.79$
Time x Genotype x Treatment	$F(2,78)=0.821$, $p=0.44$

Supplementary Table S6. Detailed statistical evaluation for Fig. 5.

One-way ANOVA repeated measures for M3G-induced hyperalgesia.

WT	Heat
Treatment	F(1, 18)=11.90, p<0.01
Time<br>Time x Treatment	F(3,54)=1.435, p=0.24<br>F(3.54)=6.582, p<0.001

WT	Mechanical
Treatment	F(1, 18)=16.76, p<0.001
Time<br>Time x Treatment	F(3,54)=0.912, p=0.44<br>F(3.54)=4.389, p<0.01

KO	Heat
Treatment	F(1, 17)=2.01, p=0.17
Time<br>Time x Treatment	F(3,51)=0.320, p=0.81<br>F(3.51)=0.486, p=0.69

KO	Mechanical
Treatment	F(1, 17)=0.341, p=0.56
Time<br>Time x Treatment	F(3,51)=0.059, p=0.98<br>F(3.51)=0.100, p=0.95

Flox	Mechanical
Treatment	F(1, 12)=0.678, p=0.42
Time<br>Time x Treatment	F(3,36)=2.455, p=0.07<br>F(3.36)=2.638, p=0.06

Flox	Heat
Treatment	F(1, 11)=1.447, p=0.25
Time<br>Time x Treatment	F(3,33)=2.138, p=0.11<br>F(3.33)=1.785, p=0.16

cKO	Mechanical
Treatment	F(1, 14)=13.016, p<0.01
Time<br>Time x Treatment	F(3,42)=2.110, p=0.11<br>F(3.42)=3.325, p<0.05

cKO	Heat
Treatment	F(1, 14)=3.849, p=0.06
Time<br>Time x Treatment	F(3,42)=2.055, p=0.12<br>F(3.42)=5.196, p<0.05

Supplementary Table S7. Detailed statistical evaluation for Supplementary Fig. S2.

One-way ANOVA repeated measures in WT or KO for analgesia and tolerance to analgesia

WT	Heat
Treatment	$F(1,18)=0.379$, $p=0.54$
Time	$F(2,36)=36.394$, $p<0.001$
Time x Treatment	$F(2,36)= 15.04$, $p<0.001$

KO	Heat
Treatment	$F(1,7)=0.34$, $p=0.57$
Time	$F(2,14)=2.057$, $p=0.12$
Time x Treatment	$F(2,14)= 1.99$, $p=0.15$

Two-way repeated measures ANOVA for hyperalgesia

WT vs KO	Heat
Genotype	$F(1, 33)=0.059$, $p=0.81$
Treatment	$F(1, 33)=4.84$, $p<0.05$
Genotype x Treatment	$F(1, 33)=2.63$, $p=0.11$
Time	$F(1,33)=15.068$, $p<0.001$
Time x Genotype	$F(1,33)=5.897$, $p<0.05$
Time x Treatment	$F(1,33)=4.119$, $p=0.051$
Time x Genotype x Treatment	$F(1,33)=19.825$, $p<0.001$

WT vs KO	Cold
Genotype	$F(1, 33)=5.21$, $p<0.05$
Treatment	$F(1, 33)=1.16$, $p=0.28$
Genotype x Treatment	$F(1, 33)=3.83$, $p<0.05$
Time	$F(1,33)=3.059$, $p=0.08$
Time x Genotype	$F(1,33)=2.080$, $p=0.15$
Time x Treatment	$F(1,33)=1.289$, $p=0.051$
Time x Genotype x Treatment	$F(1,33)=19.825$, $p<0.01$

Supplementary table S8. Detailed statistical evaluation for Supplementary Fig. S3.

ANOVA repeated measures for TLR4 mRNA expression in KO and WT mice

WT vs KO	TLR4
Genotype	$F(1, 45)=0.15$, $p=0.69$
Treatment	$F(1, 45)=2.89$, $p=0.09$
Genotype x Treatment	$F(1, 45)=0.02$, $p=0.88$

One way ANOVA for MOR mRNA expression in WT mice

WT morphine vs WT saline	MOR
Treatment	$F(1, 8)=0.39$, $p=0.54$
